# Supplementary material for: Consumer Perception of Freshness and Volatile Composition of Fresh Gilthead Seabream and Seabass in Active Packaging with and without CO2-Emitting Pads
Source: Foods. 2023 Jan 21;12(3):505. doi: 10.3390/foods12030505 (PMC9914307; doi:10.3390/foods12030505)
Supplement: Supplementary file 1 [file foods-12-00505-s001.zip › foods-2087146-supplementary.pdf]

Supplementary Materials

Table S1. Questionnaire attribute scores derived from consumer test, presented as means ± standard deviation, for whole-gutted seabream and seabass at high quality and end of high-quality shelf-life.

	Seabream (whole fish)						Seabass (whole fish)					
	High Quality			End of High Quality			High Quality			End of High Quality		
	MAP-PAD	MAP	*p-value*	MAP-PAD	MAP	*p-value*	MAP-PAD	MAP	*p-value*	MAP-PAD	MAP	*p-value*
Fresh Odor	3.57 ± 0.95	3.35 ± 1.12	0.219	3.35 ± 0.94	2.84 ± 1.14	0.004*	3.85 ± 0.87	3.71 ± 0.85	0.385	3.45 ± 0.88	3.26 ± 1.10	0.479
Bright Skin	2.08 ± 0.61	2.04 ± 0.69	0.731	2.03 ± 0.62	1.94 ± 0.64	0.414	2.15 ± 0.63	2.20 ± 0.55	0.632	2.07 ± 0.58	2.10 ± 0.58	0.757
Convex - Shiny Eyes	2.14 ± 0.64	2.21 ± 0.66	0.459	2.31 ± 0.61	2.16 ± 0.59	0.143	2.31 ± 0.64	2.21 ± 0.66	0.373	2.25 ± 0.61	2.27 ± 0.64	0.812
Coherent Flesh	1.96 ± 0.71	1.86 ± 0.70	0.411	2.04 ± 0.68	1.85 ± 0.74	0.112	2.19 ± 0.71	2.04 ± 0.65	0.166	2.21 ± 0.62	2.18 ± 0.58	0.730
Overall Freshness	3.25 ± 1.02	3.09 ± 1.14	0.369	3.28 ± 0.90	2.79 ± 1.15	0.011*	3.56 ± 0.96	3.51 ± 0.97	0.702	3.25 ± 0.87	3.18 ± 1.02	0.996
Purchase Intention	3.53 ± 1.25	3.32 ± 1.29	0.343	3.68 ± 1.04	3.18 ± 1.26	0.018*	3.73 ± 1.19	3.90 ± 1.10	0.396	3.68 ± 1.11	3.56 ± 1.32	0.753
Close to Ideal	3.22 ± 1.04	3.11 ± 1.20	0.614	3.28 ± 0.93	2.78 ± 1.13	0.009*	3.55 ± 1.18	3.60 ± 1.11	0.824	3.38 ± 1.07	3.25 ± 1.06	0.646

P-values were calculated by applying Mann-Whitney U test at a = 0.05. Significant differences at a=0,05 are depicted with (*). The attributes fresh odor, overall freshness, purchase intention, and close to ideal were scored on a 5-point scale, while bright skin, convex-shiny eyes, and coherent flesh were scored on a 3-point scale.

Table S2. Questionnaire attribute scores derived from consumer test, presented as means ± standard deviation, for fillets of seabream and seabass at high quality and end of high-quality shelf-life.

	Seabream (fillets)						Seabass (fillets)					
	High Quality			End of High Quality			High Quality			End of High Quality		
	MAP-PAD	MAP	*p-value*	MAP-PAD	MAP	*p-value*	MAP-PAD	MAP	*p-value*	MAP-PAD	MAP	*p-value*
Fresh Odor	3.66 ± 0.93	3.70 ± 0.87	0.793	3.16 ± 0.86	3.06 ± 0.97	0.544	3.77 ± 0.79	3.63 ± 0.81	0.402	3.40 ± 0.80	3.04 ± 0.91	0.030*
Flesh	2.34 ± 0.67	2.30 ± 0.71	0.740	2.19 ± 0.74	1.99 ± 0.71	0.091†	2.53 ± 0.62	2.48 ± 0.64	0.677	2.55 ± 0.56	2.38 ± 0.58	0.071†
Bright Skin	2.08 ± 0.64	2.10 ± 0.59	0.916	2.00 ± 0.53	2.00 ± 0.50	1.000	2.20 ± 0.60	2.12 ± 0.56	0.454	2.03 ± 0.55	2.00 ± 0.54	0.735
Overall Freshness	3.52 ± 1.13	3.48 ± 1.07	0.671	3.15 ± 1.03	3.00 ± 1.03	0.382	3.73 ± 0.91	3.59 ± 0.83	0.284	3.51 ± 0.86	3.18 ± 0.92	0.028*
Purchase Intention	3.78 ± 1.27	3.75 ± 1.17	0.719	3.45 ± 1.03	3.19 ± 1.05	0.205	3.97 ± 1.02	3.94 ± 0.97	0.764	3.79 ± 0.93	3.42 ± 1.08	0.045*
Close to Ideal	3.52 ±1.23	3.34 ± 1.12	0.336	3.03 ± 1.08	2.98 ± 1.05	0.846	3.77 ± 1.00	3.57 ± 1.03	0.249	3.53 ± 0.95	3.08 ± 0.99	0.010*

P-values were calculated by applying Mann-Whitney U test at a = 0.05. Significant differences at a=0,05 are depicted with (*). Tendencies (p<0.1) are depicted with (†). The attributes fresh odor, overall freshness, purchase intention, and close to ideal were scored on a 5-point scale, while flesh and bright skin were scored on a 3-point scale.

Table S3. Questionnaire attribute scores derived from consumer test, presented as means ± standard deviation, for cooked samples of seabream and seabass at high quality and end of high-quality shelf-life.

	Seabream (fillets)						Seabass (fillets)					
	High Quality			End of High Quality			High Quality			End of High Quality		
	MAP-PAD	MAP	*p-value*	MAP-PAD	MAP	*p-value*	MAP-PAD	MAP	*p-value*	MAP-PAD	MAP	*p-value*
Fresh Odor	3.86 ± 0.93	3.75 ± 0.98	0.507	3.43 ± 0.94	3.44 ± 1.10	0.888	3.77 ± 0.94	3.61 ± 1.02	0.389	3.61 ± 0.85	3.51 ± 0.94	0.594
Pleasant/Fresh Flavor	2.37 ± 0.75	2.29 ± 0.79	0.600	2.15 ± 0.77	2.15 ± 0.78	0.980	2.47 ± 0.67	2.34 ± 0.74	0.362	2.34 ± 0.73	2.23 ± 0.78	0.405
Juiciness	2.57 ± 0.59	2.53 ± 0.56	0.606	2.31 ± 0.70	2.42 ± 0.63	0.428	2.75 ± 0.44	2.65 ± 0.54	0.349	2.63 ± 0.55	2.51 ± 0.61	0.256
Overall Freshness	3.77 ± 0.91	3.55 ± 0.90	0.131	3.36 ± 0.88	3.45 ± 0.79	0.814	3.90 ± 0.84	3.67 ± 0.87	0.095†	3.68 ± 0.83	3.52 ± 0.89	0.263
Liking	5.58 ± 1.42	5.38 ± 1.48	0.430	5.07 ± 1.47	5.22 ± 1.30	0.708	5.78 ± 1.19	5.54 ± 1.34	0.276	5.64 ± 1.22	5.31 ± 1.29	0.133

Tendencies ($p<0.1$) are depicted with (†). The attributes fresh odor and overall freshness were scored on a 5-point scale, flavor and juiciness on a 3-point scale, and liking on a 7-point scale. P-values were calculated by applying Mann-Whitney U test at a = 0.05.

Table S4. Perceived occurrence of liquid in the sample packages expressed as proportion (%) of tested packages.

Fish processing	Species	Shelf-life time point	MAP-PAD (%)	MAP (%)	*p*-value
Whole fish	Seabream	HQ	10.0	45.1	<0.001
		End	9.4	55.7	<0.001
	Seabass	HQ	14.5	49.3	<0.001
		End	27.7	67.2	<0.001
Fish fillets	Seabream	HQ	14.1	39.1	0.001
		End	22.4	58.2	<0.001
	Seabass	HQ	6.3	45.3	<0.001
		End	10.4	67.2	<0.001

P-values were calculated by applying a Chi-square test at a = 0.05.

Table S5. Perceived occurrence of thin and transparent mucus on the skin of whole gutted fish in the packaging expressed as proportion (%) of tested packages.

Species	Shelf-life time point	MAP-PAD (%)	MAP (%)	*p*-value
Seabream	HQ	33.3	42.7	0.239
	End	41.8	45.6	0.657
Seabass	HQ	40.0	44.0	0.620
	End	41.2	55.9	0.086

P-values were calculated by applying a Chi-square test at a = 0.05.

Table S6. Proportion (%) of consumers who perceived cooked seabream and seabass samples as firm in texture.

Species	Shelf-life time point	MAP-PAD (%)	MAP (%)	*p*-value
Seabream	HQ	69.6	77.3	0.408
	End	91.8	80.0	0.091
Seabass	HQ	62.2	61.7	0.959
	End	78.3	82.4	0.612

P-values were calculated by applying a Chi-square test at a = 0.05.

Table S7. Mean concentrations (ng/g) and standard deviations (SD) of the volatile compounds that differed significantly (p<0.05) or showed a tendency for difference (p<0.1) in seabream fillets. P-values were calculated by applying a mixed model ANOVA (factors: pad, shelf-life, pad × shelf-life).

	2-way ANOVA			High Quality				End of High Quality			
	Pad	Shelf-life	Pad × Shelf-life	MAP-PAD		MAP		MAP-PAD		MAP	
				Mean	SD	Mean	SD	Mean	SD	Mean	SD
**Alcohols**											
2-penten-1-ol	ns*	0.067	0.052	11.33	4.17	8.26	3.27	11.05	1.63	16.17	2.82
1-octen-3-ol	ns	0.056	ns	14.59	4.05	9.69	4.19	19.38	6.10	19.62	7.73
1-octanol	0.085	ns	ns	1.80	0.61	0.92	0.08	1.84	0.81	1.55	0.15
(E,E)-3,5-octadien-3-ol	ns	ns	0.094	3.75	1.65	2.27	0.26	3.27	1.19	4.63	1.58
2-octen-1-ol	ns	0.058	ns	1.44	0.63	1.05	0.38	2.11	0.76	2.36	0.92
**Aldehydes**											
Hexanal	ns	0.085	ns	48.48	8.41	37.44	18.94	51.15	11.85	67.03	15.55
Heptanal	0.085	ns	0.047	10.41	1.15	5.58	1.27	9.48	1.90	10.02	1.26
Z-4-heptenal	ns	0.073	ns	5.04	1.88	4.03	1.82	5.94	1.39	7.85	2.63
Octanal	ns	ns	0.02	8.31	1.97	5.26	1.01	5.76	1.49	8.07	0.83
**Alkanes**											
Undecane	ns	ns	0.009	7.99	0.81	5.93	1.06	6.74	1.78	9.51	1.07
**Aromatic hydrocarbons**											
Ethylbenzene	ns	ns	0.006	9.87	0.63	7.02	1.73	7.90	1.45	11.95	2.20
**Esters**											
Ethyl acetate	ns	<0.001	ns	42.26	16.49	29.35	6.91	6.31	1.53	5.29	1.17
**Ketones**											
2,3-pentanedione	ns	0.011	ns	11.30	1.13	10.35	5.76	15.00	4.45	21.81	3.01
2-butanone, 3-hydroxy-	ns	0.058	ns	2.93	0.93	2.69	1.23	8.18	4.89	31.40	21.74

*ns stands for not significant effect

Table S8. Mean concentrations (ng/g) and standard deviations (SD) of the volatile compounds that differed significantly ($p<0.05$) or showed a tendency for difference ($p<0.1$) in seabass fillets. P-values were calculated by applying a mixed model ANOVA (factors: pad, shelf-life, pad × shelf-life).

	2-way ANOVA			High Quality				End of High Quality			
	Pad	Shelf-life	Pad × Shelf-life	MAP-PAD		MAP		MAP-PAD		MAP	
				Mean	SD	Mean	SD	Mean	SD	Mean	SD
**Alcohols**											
1 penten-3-ol	0.021	0.001	0.013	11.11	3.07	10.43	4.29	14.73	3.49	27.32	3.41
1-octanol	ns*	0.086	ns	1.87	0.37	2.39	0.82	1.41	0.25	1.75	0.30
**Aldehydes**											
Hexanal	0.042	ns	ns	31.88	4.11	34.63	5.49	26.42	4.13	40.07	8.63
Octanal	ns	0.069	ns	7.43	0.53	8.98	1.36	6.20	0.99	6.89	2.08
Decanal	ns	ns	0.096	22.43	11.64	30.08	6.10	29.28	8.35	19.79	2.13
**Alkanes**											
2,2,4,6,6-pentamethylheptane	ns	ns	0.085	24.93	15.79	10.04	6.32	14.13	5.88	25.60	14.74
Undecane	0.061	0.078	ns	9.69	1.04	9.96	0.44	7.93	1.07	9.76	0.61
Pentadecane	ns	0.048	ns	3.90	1.72	1.92	0.82	4.89	1.40	5.64	1.34
**Aromatic hydrocarbons**											
P-xylene	0.058	ns	ns	5.29	0.97	7.25	1.01	5.62	1.40	7.25	2.00
**Esters**											
Acetic acid methyl ester	ns	0.062	0.031	5.09	0.16	4.32	1.09	4.77	1.64	7.62	0.78
Butanoic acid, methyl ester	ns	0.035	ns	9.35	0.66	8.82	1.18	10.63	0.82	11.26	1.98
**Ketones**											
Acetone	ns	0.016	0.072	5.57	0.46	4.46	1.47	6.22	1.11	7.82	0.62
2,3-pentanedione	0.052	0.025	ns	5.21	0.14	6.16	1.95	6.83	1.37	12.15	4.13
5-hepten-2-one, 6- methyl	ns	0.085	0.096	4.53	1.24	3.29	1.65	4.58	0.80	6.05	1.13
**Terpenes**											
Alpha pinene	ns	0.049	ns	5.08	0.34	4.74	1.87	5.75	0.83	7.27	1.19

*ns stands for not significant effect

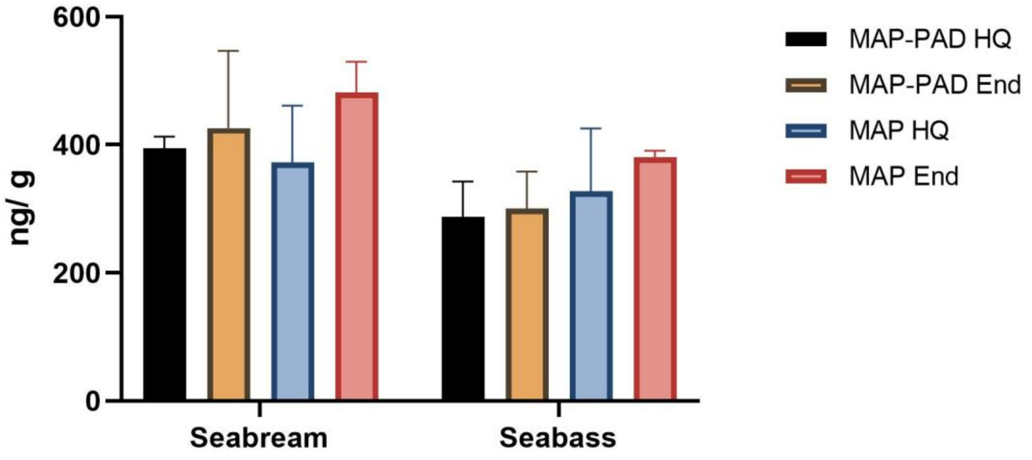

Figure S1. Total concentration (ng/g) of volatile compounds identified via HS-SPME-GC-MS analysis in seabream and seabass fillets packed in active packaging with (MAP-PAD) or without (MAP) a pad at high quality (HQ) or the end of high-quality (End) shelf-life.

Table S9. K-values (%) derived from the ATP-breakdown analysis, presented as means ± standard deviation, for whole-gutted and filleted sea bream and seabass at high quality and end of high-quality shelf-life.

Fish processing	Species	Shelf-life time point	MAP-PAD	MAP	*p*-value
Whole fish	Sea bream	HQ	14.7 ± 2.6	14.7 ± 2.6	0.749
		End	25.4 ± 2.2	26.4 ± 1.8	0.522
	Seabass	HQ	35.6 ± 4.9	40.4 ± 5.7	0.262
		End	55.8 ± 7.6	52.9 ± 5.7	0.522
Fish fillets	Sea bream	HQ	21.7 ± 1.8	21.2 ± 1.7	0.522
		End	30.7 ± 3.6	28.1 ± 3.3	0.200
	Seabass	HQ	46.9 ± 2.6	44.5 ± 2.6	0.262
		End	58.8 ± 3.7	59.9 ± 3.7	0.522

P-values were calculated by applying Mann-Whitney U test at a = 0.05.

Table S10: Bacterial population, expressed in log CFU g^{-1}, of total viable count, *Pseudomonas* spp., *Enterobacteriaceae* spp. and *Shewanella* spp. for whole-gutted and filleted gilthead seabream and seabass at the end of high-quality shelf-life time point.

Fish processing	Species	Packaging	Total Viable Count	*Pseudomonas* spp.	*Enterobacteriaceae* spp.	*Shewanella* spp.
Whole fish	Seabream	MAP-PAD	5.4 ± 0.2	5.6 ± 0.1	3.4 ± 0.2	7.2 ± 0.2
		MAP	6.2 ± 0.1	4.5 ± 0.1	4.0 ± 0.2	5.8 ± 0.2
	Seabass	MAP-PAD	6.2 ± 0.2	5.3 ± 0.1	4.7 ± 0.2	5.8 ± 0.2
		MAP	6.8 ± 0.1	5.5 ± 0.1	5.0 ± 0.1	6.7 ± 0.2
Fish fillets	Seabream	MAP-PAD	4.9 ± 0.6	4.2 ± 0.2	3.0 ± 0.5	4.5 ± 0.1
		MAP	6.4 ± 0.2	4.4 ± 0.6	3.9 ± 0.3	5.9 ± 0.3
	Seabass	MAP-PAD	4.9 ± 0.3	4.4 ± 0.1	2.3 ± 0.3	4.6 ± 0.3
		MAP	5.3 ± 0.9	4.8 ± 0.8	3.3 ± 0.6	5.4 ± 0.4
